# Attractiveness of Human Faces: Norms by Sex, Sexual Orientation, Age, Relationship Stability, and Own Attractiveness Judgements

**DOI:** 10.3389/fpsyg.2020.00419

**Published:** 2020-03-13

**Authors:** Josefa N. S. Pandeirada, Natália Lisandra Fernandes, Marco Vasconcelos

**Affiliations:** ^1^Centro de Investigação em Tecnologias e Serviços de Saúde, University of Aveiro, Aveiro, Portugal; ^2^William James Center for Research, University of Aveiro, Aveiro, Portugal

**Keywords:** human faces, face attractiveness, normative data, online vs. laboratory data collection, cultural agreement

Facial attractiveness (FA) impacts various aspects of human life making it widely studied. This study presents attractiveness norms for a set of 223 neutral faces of young adults collected both online and in the laboratory. Furthermore, data are presented according to variables known to influence attractiveness judgments: sex, sexual orientation, age, and relationship status of the respondent. Finally, our data can be used to study FA across cultures and is possibly useful to researchers studying FA in other cultures. The potential impact of these norms is as large as the variety of domains in which attractiveness is relevant.

## Introduction

Human faces are an extremely important vehicle of information about individuals. Attractiveness, in particular, is one of the most relevant face characteristics influencing human interaction in various ways. Its importance has been recognized in socialization and evolutionary theories and is well-summarized by Langlois et al. (2000): “the effects of facial attractiveness are robust and pandemic, extending beyond initial impressions of strangers to actual interactions with those whom people know and observe” (p. 404). Indeed, the importance of facial attractiveness (FA) has been demonstrated across various fields, as briefly reviewed next.

In the domain of mate choice, FA has been linked to genetic fitness thus suggesting that a preference for attractive faces may represent an adaptive mechanism that ultimately promotes fitness (e.g., Jokela, 2009; Pflüger et al., 2012). Accordingly, several studies have found a positive relation between men's FA and overall genetic quality (e.g., Roberts et al., 2005), and an enhanced and more efficient immune functioning (Rantala et al., 2012); in women, FA relates to features of long-term health and fertility (e.g., Rantala et al., 2013; but see Cai et al., 2019).

In the social domain, some studies have reported a positive relation between attractiveness and socially desirable personality traits (Lorenzo et al., 2010), such as trustworthiness (e.g., Wilson and Eckel, 2006; but see Olivola and Todorov, 2017), intelligence (Kanazawa, 2011), agreeableness, and extraversion (Meier et al., 2010). Further, people seem to assign more positive characteristics to attractive people (“beautiful is good”), but also negative characteristics to the less attractive (“ugly is bad”) (Griffin and Langlois, 2006).

Attractiveness seems to influence both our perception of individuals and our behavior toward them. In academia, teachers consider more attractive students as more intelligent and as having more academic potential and social skills (Ritts et al., 1992); these students also tend to receive higher grades. On the other side, students rate more attractive teachers more highly in terms of quality, helpfulness, and clarity (Bonds-Raacke and Raacke, 2007); these teachers also seem to get students more engaged and motivated in their courses (Liu et al., 2013).

In the job market, attractive job applicants are generally perceived as more qualified, are more likely to obtain favorable hiring recommendations, have a job, receive job promotions, and secure higher wages (e.g., Pfeifer, 2011). Regarding companies, Pfann et al. (2000) found higher profits and growth success in advertising firms that had more attractive executives compared to those with less attractive executives. Even leadership relates to attractiveness in many ways (for an overview, see Poutvaara, 2014).

Within the justice system, attractiveness influences judgments of culpability. For example, more attractive individuals seem to benefit from a leniency bias in crimes such as robbery, but are likely to be treated more severely in crimes such as negligent homicide (Mazzella and Feingold, 1994). On the victim's side, the perception of injustice of the crime, as well as the level of punishment inflicted to the perpetrator, tend to be higher for more attractive female victims (Callan et al., 2007).

Finally, FA also affects various cognitive processes, including attention (for a review, see Lindell and Lindell, 2014), memory (e.g., Wiese et al., 2014), and even has implications in brain activity (Hahn and Perrett, 2014; Siuda et al., 2015). It also seems to have a neural processing time course different from the perception of other attributes (Calvo et al., 2018).

This brief overview highlights the relevance of studying FA. Despite the probable contribution of other factors (e.g., trustworthiness) to the aforementioned phenomena (Todorov et al., 2015; Olivola and Todorov, 2017), FA seems nonetheless to play a key role thus stressing the need for properly normed facial stimuli. Here we provide FA ratings for more than 200 faces from a relatively heterogeneous sample of participants. Even though data collection via the internet has increased dramatically over the last years (e.g., Denissen et al., 2010), some studies stress potential comparability issues between data collected online and data collected in the laboratory (e.g., Barenboym et al., 2010); other studies indicate that they are equivalent (e.g., Kuperman et al., 2012). Thus, we collected most of our data online but also in a laboratory setting for validation of the online data. Data are also provided according to a set of variables known to influence attractiveness ratings: the participants' sex and sexual orientation (Hahn et al., 2016; Mitrovic et al., 2016), the participants' age (e.g., Foos and Clark, 2011), and his/her involvement in a relationship (e.g., Lydon and Karremans, 2015) (see details in the data description). The data collected online are of particular interest here as these variables are better represented in this sample.

## Methods

### Participants

#### Online Sample

A total of 827 participants responded until completion to the online questionnaire (302 participants failed to complete the questionnaire). Non-Portuguese participants were excluded in order to maintain a culturally homogenous sample (*n* = 55). Data from 15 participants were also excluded due to randomization errors in the questionnaire. The final online sample included 757 participants (females = 543; 72%), aged between 18 and 75 years (*M* = 29.45, *SD* = 10.79). These data were collected between January and June of 2014.

#### Laboratory Sample

Data from 117 students were collected in the laboratory using the same online questionnaire. Data from eight participants were excluded due to non-Portuguese nationality and from other three due to questionnaire running errors. This final sample includes 106 participants (female = 71; 67%) aged between 18 and 51 years old (*M* = 21.87, *SD* = 6.09). These participants were either volunteers or participated in exchange for course credits. Data were collected at the Universities of Aveiro, Coimbra, and Minho between December of 2013 and July of 2014 under similar laboratory conditions.

Table 1 reports a complete characterization of the samples regarding sex, age group, sexual orientation, and relationship stability. Given that sexual orientation has been reported as potentially relevant in FA assessments (e.g., Mitrovic et al., 2016), and given the low representativeness of non-heterosexuals in our samples (including homosexual, bisexual, and participants with “other sexual orientation”), we only consider the data from the heterosexual participants when reporting the data broken down by the other variables. Heterosexual participants corresponded to 91.8 and 97.1% (*n* = 695 and *n* = 103) of the online and laboratory samples, respectively.

**Table 1:** Characterization of the heterosexual online and laboratory samples regarding sex, age group, and relationship stability.

	**Online sample**		**Laboratory sample**	
	***n***	**Percentage**	***n***	**Percentage**
**Gender**				
Female	508	73.1	69	67.0
Male	187	26.9	34	33.0
Age group^*^				
Young adults	420	60.4	94	91.3
Middle-aged adults	220	31.7	7	6.8
Older adults	55	7.9	2	1.9
**Relationship stability**				
In a stable relationship	431	62	51	49.5
Not in a stable relationship	264	38	52	50.5

* *Following McLellan and McKelvie (1993) the age groups were defined as follows: Young adults: 18–29 years; Middle-aged adults: 30–49 years; Older adults: ≥50 years*.

### Material

The stimuli consisted of 223 frontal-view, colored young adult facial photographs (122 males and 101 females), displaying direct eye gaze and a neutral facial expression. Although there is evidence for cross-cultural consistency in judgments of FA, this can be influenced by familiarity and perceptual experience with a specific group of faces (e.g., Coetzee et al., 2014); therefore, we decided to use databases containing faces similar to those of the Portuguese population. Faces were further selected by two of the authors according to this last criterion, along with the information regarding the ethnicity of the face stimuli available in some of the databases. When selecting the databases, the following inclusion criteria were also used: (1) photographs were taken under controlled conditions (such as illumination setting and uniform background); (2) participants used a standard t-shirt and removed jewelry, glasses, and makeup; and, (3) they corresponded to faces of young adults.

Our study included faces from the following databases: (1) Karolinska Directed Emotional Faces (KDEF; Lundqvist et al., 1998); (2) Warsaw Set of Emotional Facial Expression Pictures (WSEFEP; Olszanowski et al., 2014); (3) Radboud Facial Database (RaFD; Langner et al., 2010); (4) FACES Database (Ebner et al., 2010); and, (5) Amsterdam Dynamic Facial Expression Set (ADFES; Van Der Schalk et al., 2011). Written permission for use was obtained from their corresponding authors and/or laboratories. When applicable, written consent was also obtained to edit the photos in order to obtain more homogeneous stimuli across the selected databases. Specifically, a standardized neutral-white background was applied to all stimuli and the size of the images was adjusted to 337 × 457 (l × h) pixels, the size of the images from the KDEF. In particular, images from the FACES database were reduced to 22% of their original size, and the ADFES, and Warsaw pictures were increased to 125% and 136% of their original size, respectively. Additionally, the central point of the image was set to be at the crossing point between the central vertical and horizontal lines of the face. The tonality and color balance of images from the KDEF database were also adjusted to provide higher homogeneity across images; specifically, images were adjusted for brightness and contrast (set to 49 and −32, respectively), saturation and vibrance (set to −16 and 13, respectively), levels (shadows: 22; midtones: 1.66) and curves (output: 102; input: 81). Editing was done using *Adobe Photoshop CC*. From the original set of images, 14 photographs were excluded due to one of the following: (a) the top of the head was not visible in the picture; (b) facial characteristics were too different from the Portuguese population (according to the procedure previously described), or; (c) we were unable to edit the photograph to make it homogenous with the remaining stimulus set.

Each participant evaluated only 50 of the faces to avoid a very lengthy task. The to-be-rated faces were pseudo-randomly selected from the total set of stimuli with the following constraints: (1) the same number of female (*n* = 25) and male faces (*n* = 25) was presented in each questionnaire, and; (2) the number of faces selected from a given database was proportionally similar for all databases. The final distribution of stimuli by database, as well as the number of stimuli from each database, is presented at the bottom of the “read me first” tab of the data file available at https://osf.io/vudr2/?view_only=0e732a0add6149069aa7c26aa57cba4f.

### Procedure

A questionnaire was prepared using the software LimeSurvey. The questionnaire, as well as the data, was housed in a local server at the University of Aveiro. For the online data collection, a brief description of the questionnaire was sent by e-mail to various recipients for dissemination (e.g., universities, professional schools, and other large companies across the country) along with the electronic link to access the questionnaire. For the laboratory data collection, participants were recruited at the Universities of Aveiro, Minho, and Coimbra. Participants were required to be at least 18 years old; no other exclusion criteria were presented. All participants responded to the same questionnaire.

The opening page of the questionnaire provided a brief description of the study, confidentiality information, and an informed consent request. If no consent was granted, participants were thanked and the program ended; otherwise, the program moved on to collect the following socio-demographic information: sex, age, nationality, sexual orientation, marital status^1^, and whether they were in a stable relationship. The attractiveness rating procedure then followed. The initial instructions informed participants they would be shown faces sequentially and that they would be rating how attractive each face was to them using a 7-point rating scale, where 1 corresponded to “not attractive at all,” and 7 to “very attractive.” Participants were given unlimited time to respond to each face but were instructed to respond quickly and to rely on their “gut instinct.” They were also told that their answers represented their personal view and that there were no correct or incorrect responses.

The 50 faces were then presented one at a time on a white background in a randomized order for each participant. Each face was presented at the center of the screen with the response scale below it; this was represented by a series of radio buttons along with the labels for the values 1 and 7. Responses were mandatory and implemented by selecting the radio button that corresponded to the value of the participant's choice. Each picture was preceded by a 1,000-ms fixation cross and followed by a 500-ms blank screen.

After rating all the faces, participants were asked to rate their own attractiveness (i.e., how attractive they considered themselves to be) and how attractive they thought other people would rate them. The presentation procedure used for the face stimuli was followed except that the actual face was replaced by a shadow image of a human face. The questionnaire ended here for the male participants but additional data not relevant to the current study were collected for the female participants^2^. A final appreciation message was presented at the end.

## Results

### Overall Data

Each face was rated by about 170 (*SD* = 22; range: 115–227) and 24 participants (*SD* = 5; range: 13–38) from the online and laboratory samples, respectively. This variation in the number of ratings was due to the pseudo-random stimuli-selection procedure implemented for each participant (see details in the Material description); while not ideal, this procedure introduced variation both in the set of faces rated by each participant and in their order of presentation, thus minimizing possible face order and group effects on ratings. In some cases, the mean number of observations per face was either low or missing. Therefore, we opted not to present the data when the mean number of observations per face was lower than 5; these cases are noted in each of the tabs of the data file. Detailed information about the ratings obtained from each sample is provided in the data file available at https://osf.io/vudr2/?view_only=0e732a0add6149069aa7c26aa57cba4f. The file includes the following tabs and content referring to the data collected online and in the laboratory:

1. Read me first: Describes the information presented in each of the tabs. The number of faces presented from each database in each questionnaire is also provided at the bottom of this tab;
2. Overall Data: This tab presents the mean attractiveness rating values for the overall samples;
3. Sexual Orientation: Mean attractiveness ratings (and SDs) are presented according to sexual orientation. The online data are presented for the heterosexual, homosexual, and bisexual participants. The data collected in the laboratory are presented for the heterosexual participants only;
4. Age Group: Mean attractiveness ratings (and SDs) of each face are presented for three age groups following the criteria from McLellan and McKelvie (1993): “young-adult raters” (18–29 years), “middle-aged raters” (30–49 years), and “old raters” (≥50 years). Data from the online sample are presented for each of these groups, whereas those from the laboratory are presented only for the “young-adult raters.”
5. Relationship Stability: Mean attractiveness ratings (and SDs) for each face according to the participants' involvement in a stable relationship: “involved in a stable relationship” or “not involved in a stable relationship”;
6. Sex and other variables: Mean attractiveness ratings (and SDs) of each face are presented by sex (females and males). These data are further broken down by the following variables (as described above): sexual orientation, age groups, and relation stability;
7. Self and Others Evaluation: Mean attractiveness ratings (and SDs) are presented for each face according to the participants' self-perceived attractiveness as well as according to what the participant thinks others perceive of his/her attractiveness. The original responses were recoded to create three groups: low (ratings of 1–2), average (ratings of 3–5), and high attractiveness (ratings of 6–7).

Across all the data tabs, column A indicates the source database. The face reference reported in column B corresponds to the labeling of the face in our questionnaire and in column C in its original database. In all datasets, the “N” indicates the number of participants that contributed to the presented attractiveness mean and standard deviation that follow. Table 2 reports the mean number of ratings and mean attractiveness values per face, and separately for the female and male faces. Data from the online and laboratory samples are provided as well according to the variables of interest.

**Table 2:** Mean number of responses and mean attractiveness ratings, and corresponding Standard Deviations (SD), for each face, and separately for the female and male faces.

**Raters**	**All faces**				**Female faces**				**Male faces**			
	**Mean number of responses (SD)**		**Mean attractiveness (SD)**		**Mean number of responses (SD)**		**Mean attractiveness (SD)**		**Mean number of responses (SD)**		**Mean attractiveness (SD)**	
	**Online**	**Lab**	**Online**	**Lab**	**Online**	**Lab**	**Online**	**Lab**	**Online**	**Lab**	**Online**	**Lab**
Full sample	169.73 (21.87)	23.77 (5.11)	2.92 (0.81)	2.85 (0.87)	187.38 (16.45)	26.24 (4.67)	3.24 (0.81)	3.16 (0.86)	155.12 (13.37)	21.72 (4.55)	2.66 (0.73)	2.60 (0.80)
Female	113.90 (15.75)	15.47 (3.75)	2.97 (0.83)	2.86 (0.92)	125.74 (12.70)	17.08 (3.55)	3.22 (0.81)	3.11 (0.89)	104.10 (10.38)	14.14 (3.39)	2.77 (0.80)	2.65 (0.90)
Male	41.93 (7.47)	7.62 (2.74)	2.76 (0.84)	2.88 (0.90)	46.29 (6.84)	8.42 (2.62)	3.30 (0.82)	3.31 (0.89)	38.32 (5.90)	6.97 (2.68)	2.31 (0.54)	2.52 (0.74)
Young adults	94.17 (13.07)	21.08 (4.87)	2.91 (0.85)	2.81 (0.88)	103.96 (10.37)	23.27 (4.59)	3.24 (0.84)	3.11 (0.87)	86.07 (8.85)	19.26 (4.33)	2.64 (0.77)	2.56 (0.80)
Middle-aged	49.33 (8.83)	1.57 (1.16)	2.91 (0.79)	3.35 (1.32)	54.46 (7.96)	1.73 (1.20)	3.23 (0.79)	3.70 (1.11)	45.08 (7.10)	1.43 (1.12)	2.65 (0.68)	3.03 (1.41)
Older	12.33 (3.45)	0.45 (0.59)	2.97 (0.79)	3.65 (1.19)	13.61 (3.26)	0.50 (0.64)	3.28 (0.78)	3.96 (1.06)	11.27 (3.25)	0.41 (0.54)	2.72 (0.70)	3.36 (1.25)
With a stable relationship	96.64 (13.85)	11.43 (3.30)	2.84 (0.81)	2.83 (0.93)	106.68 (11.53)	12.62 (3.34)	3.18 (0.80)	3.18 (0.91)	88.32 (9.38)	10.45 (2.95)	2.56 (0.71)	2.53 (0.85)
Without a stable relationship	59.19 (8.98)	11.66 (3.36)	3.04 (0.83)	2.88 (0.90)	65.35 (7.13)	12.87 (3.32)	3.34 (0.83)	3.15 (2.66)	54.10 (6.95)	10.66 (3.06)	2.80 (0.75)	2.66 (0.86)

*Data from the online and laboratory samples are presented for the full sample and according to the variables of interest*.

## Discussion

This study provides attractiveness ratings for a large set of faces collected both online and in the laboratory. The influence of attractiveness is undeniable in various domains as briefly reviewed in the Introduction justifying the need to make such normative data widely available. Besides collecting attractiveness ratings, we also gathered information on a set of variables of potential interest thus opening new opportunities to explore in an integrated manner the potential role played by each of these variables in the assessment of FA. For example, few studies have explored how these different variables interact with each other over the lifespan (Ebner et al., 2018). Additionally, by providing data for a large number of faces and separately for each of these variables, researchers will be able to use a number of criteria to select their stimuli without running the risk of ending up with a reduced set of stimuli.

Considering that normative ratings for the faces assessed in this study have been collected in other countries, one could explore possible regularities on the assessment of attractiveness across countries. One often finds research reports denoting that researchers spent resources and time conducting pilot studies to collect their own attractiveness ratings (e.g., Jones et al., 2007; Pegors et al., 2015). We tentatively calculated the consistency between our ratings and those obtained in Olszanowski et al. (2014) study for the WSEFEP database which used the same rating scale we used. The intraclass correlations for the average measures was of 0.907, 0.912, and 0.853, for the entire stimuli set, female and male faces, respectively. This strong agreement, along with the literature reporting cross-cultural agreement on attractiveness evaluations, provide initial support to the idea that our normative data could be useful for researchers in other countries and cultures. Yet, comparisons with datasets from other countries containing normative data for some of the images used in our study could be conducted to explore the potential generalization of our data (e.g., with the FACES database by Ebner et al., 2010; the corresponding norming data can be found at https://faces.mpdl.mpg.de/imeji/; or with the RaFD database by Langner et al., 2010; the corresponding datasets can be found at http://gijsbijlstra.nl/227-2/). Furthermore, comparisons could be drawn with a more recent normative study conducted in Portugal that also included some of our stimuli (e.g., Garrido and Prada, 2017; the corresponding dataset can be found at https://osf.io/fvc4m/). Such comparisons should nonetheless take into account potential methodological differences between studies that could influence the ratings collected (e.g., color vs. gray scale stimuli; single vs. multiple faces presentation; self-paced vs. timed rating; age of the participants). Also, attractiveness obtained for neutral-looking faces may differ from those obtained when they are intermixed with the same faces expressing different emotions. As reported by Garrido and Prada (2017), attractiveness ratings correlate strong and positively with valence and familiarity; one can wonder if a given neutral face is rated differently when the same face displaying a happy or angry expression was seen and rated previously.

As mentioned before, controversy remains regarding the reliability of data collected online and in the laboratory. An initial inspection of our data suggests a very high level of agreement. Specifically, the intraclass correlations for the average measures was of 0.963, 0.957, and 0.961, for the total set of stimuli, the female and male faces, respectively. Still, many aspects of this consistency remain to be explored (e.g., is this consistency different for the male / female faces or between sexes?).

Summing up, this report provides attractiveness ratings for a large set of faces and from a large sample of participants. These data were obtained from a very heterogeneous sample which allowed us to present the data according to several variables of potential interest to researchers, namely age and sex of the participant, sexual orientation, and relationship stability of the respondent; the analyses of the influence of these variables in the attractiveness ratings should be informative to the literature. Information regarding the self and other-perceived attractiveness is also provided. Our preliminary exploration suggests that researchers from other countries and cultures could also rely on these data when selecting their stimuli. Therefore, we expect our data to be of great value for researchers from the various research domains where attractiveness has been shown to play an important role.

## Data Availability Statement

The datasets are available on the Open Science Framework through the following link: https://osf.io/vudr2/?view_only=0e732a0add6149069aa7c26aa57cba4f.

## Ethics Statement

All procedures were in accordance with the 1964 Helsinki declaration and its later amendments, in particular with the following aspects: participation in the project added no risk or burden to participants, the benefits of the project clearly outlined its potential risks, written informed consent was obtained from all participants, participation was volunteer, and data were managed and are presented in a manner that insures the participants' confidentiality.

## Author Contributions

JP and NF developed the idea and procedures to collect the data. JP coordinated the data acquisition. NF managed the data collection process and provided preliminary analysis. They both drafted the manuscript which was then discussed, reviewed and commented along with MV. All authors approved the final version of this manuscript.

### Conflict of Interest

The authors declare that the research was conducted in the absence of any commercial or financial relationships that could be construed as a potential conflict of interest.

## Footnotes

^1^Information on the marital status reported by the participants is not provided due to the large frequency disparity across groups.

^2^Female participants also provided information on the average duration of their menstrual cycle, date of last menstrual cycle, and use of oral contraceptives. We aimed to determine the phase of the menstrual cycle using this information but the collected data did not allow us to do it in a systematic manner for a considerable set of participants.
